# Comparison of three adhesive systems in class II composite restorations in endodontically treated teeth: Influence of Er:YAG laser conditioning and gingival margin levels on microleakage

**DOI:** 10.4317/jced.54843

**Published:** 2018-08-01

**Authors:** Emel-Olga Onay, Kivanc Yamanel, Yonca Korkmaz-Ceyhan, Kamran Gulsahi

**Affiliations:** 1 Professor, Department of Endodontics, School of Dentistry, Baskent University, Bahcelievler-Ankara, Turkey; 2 Associate Professor, Department of Restorative Dentistry, School of Dentistry, Baskent University, Bahcelievler-Ankara, Turkey; 3 Associate Professor, Department of Restorative Dentistry and Prosthodontics, The University of Texas School of Dentistry at Houston, Houston, TX, USA

## Abstract

**Background:**

Dental surface conditioning by Er:YAG laser is currently being investigated, as not all of the mechanisms and effects of this technique have been clearly studied. Thus, the aim of the present study was to assess the cervical microleakage of Class II resin composite restorations in endodontically treated teeth following either the respective conventional conditioning or additional Er:YAG laser conditioning, in association with varied adhesives.

**Material and Methods:**

Standardized mesial-occlusal-distal cavities (two gingival walls positioned in dentin and enamel, respectively) were created in 60 extracted human premolar teeth. Following the completion of the endodontic therapy, the teeth were grouped into six categories based on conditioning modality and adhesive strategy as follows: group 1-37% phosphoric acid/Adper Single Bond 2 (ASB2); group 2-Er:YAG laser/37% phosphoric acid/ASB2; group 3-Clearfil SE Bond (CSE); group 4-Er:YAG laser/CSE; group 5-Adper Easy One (AEO); and group 6-Er:YAG laser/AEO. Specimens were submitted to thermocycling and dye penetration, followed by longitudinal sectioning. The dye penetration was evaluated using a stereomicroscope. One specimen from each group was assessed under a scanning electron microscope for adhesive interface analysis.

**Results:**

No significant differences were found between the conditioning modalities, nor between the adhesive systems at both margins. Groups 1 and 2 showed a lower degree of microleakage in the enamel vs. dentin (*p* = 0.002). Group 2 showed a significantly lower incidence of microleakage in enamel vs. dentin (*p* = 0.005).

**Conclusions:**

CSE and AEO were comparable with that of ASB2 regarding sealing ability. Additional Er:YAG laser conditioning may be beneficial before ASB2 application in enamel.

** Key words:**Endodontically treated teeth, etch-and-rinse adhesive, Er:YAG laser, gingival level, sealing ability, self-etch adhesive.

## Introduction

The effect of the concept of coronal microleakage on the endodontic treatment outcome has been known for a long time (1). The bacteria-tight seal of the endodontic access cavity plays a major role in the effectiveness of the root canal treatment. The presence of an insufficient sealing at coronal restoration margins permits oral bacteria and their metabolic products to gain entry into the filled root canal, which is a contributing factor for the progression and perpetuation of periapical pathosis (1). Moreover, in one of the studies, the operative adequacy of the coronal restoration was considered more important as compared to operative adequacy of the endodontic treatment (2).

In order to interact with the dentinal smear layer, the current adhesive systems use one of the following strategies: etch-and-rinse technique or self-etching technique. In the case of etch-and-rinse technique, phosphoric acid gel is used at a concentration of 30-40% to etch dentin and enamel prior to adhesive application. After its application, the smear layer is removed, resulting in the opening up of the dentinal tubules. In the case of the self-etching technique, a particular acid-etch step is not needed for the adhesives nor is the smear layer removed. Aqueous mixtures of functional acidic monomers with a high pH value compared to phosphoric acid etching gels are used in this step (3).

The erbium: yttrium aluminum garnet (Er:YAG) laser technique has emerged as one of the alternative techniques for safe and effective conditioning of the enamel and dentin, as irradiated dental hard tissues provide microscopically irregular surfaces on both tissues and expose dentin tubules without a smear layer (4). Previous studies have shown that a considerable improvement was observed in marginal seal integrity and durability of the adhesive restorations in terms of sealing ability when the etch-and-rinse technique was achieved following Er:YAG laser conditioning (5-7). However, the relationship between different adhesive strategies and dental surface conditioning by Er:YAG laser has not been well studied yet.

Thus, the present study aimed to evaluate the cervical microleakage of Class II resin composite restorations in endodontically treated teeth with the margins positioned apical and coronal to the cemento-enamel junction, which either followed the conventional conditioning or additional Er:YAG laser conditioning, in association with one etch-and-rinse adhesive and two self-etch adhesives.

## Material and Methods

The study used 60 extracted human premolar teeth with orthodontic indication for experimental purpose. The exclusion criteria included cracks, hypoplastic areas, restorations, and caries. The standardized Class II mesial-occlusal-distal (MOD) cavities (buccolingual width of 3 mm on the occlusal and gingival sides) were prepared with a #1090 diamond fissure bur (Diatech Dental AG, Heerbrugg, Switzerland) in a high-speed turbine under copious water spray. After every five preparations, new burs were used. The mesial gingival wall was created 1 mm coronal to the cemento-enamel junction (enamel area) while the distal gingival wall was created 1 mm apical to the cemento-enamel junction (dentinal area) in each cavity. No margins were beveled.

The removal of the pulp chamber roof exposed the root canals. Then the pulp tissue was extirpated. A step-back technique was used to shape the canal to a size of 40 master apical file. Manually used nickel-titanium files (Dentsply Maillefer, Ballaigues, Switzerland) were used for the root canal preparation. The irrigation was performed by alternating the solutions of 2.5% NaOCl and 17% EDTA after every change of instrument. After the final irrigation with distilled water, the paper points were used to dry the canals, which were then filled with lateral condensation technique using gutta-percha (Diadent, Chongju, Korea) and AH Plus sealer (Dentsply De Trey GmbH, Konstanz, Germany). A conventional glass ionomer cement (Riva Self Cure; SDI Ltd., Bayswater, Victoria, Australia) was used for the sealing purpose of the coronal root canal openings. The samples were grouped into six groups with 10 teeth each (T Table 1).

**Table 1:**
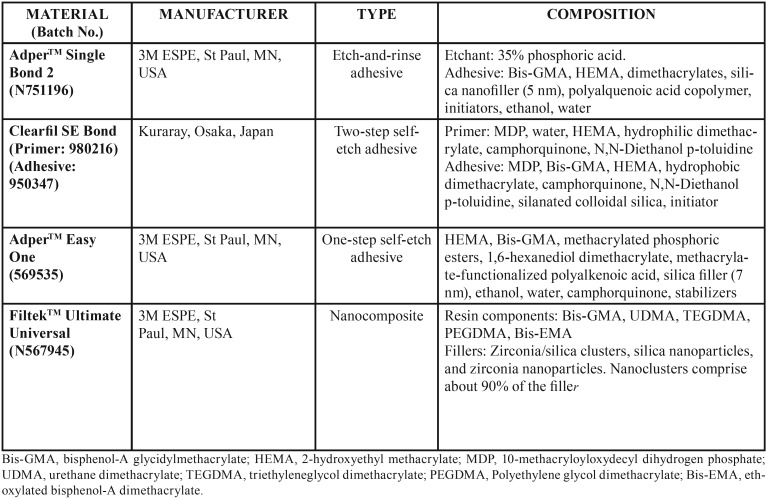
Chemical composition of the materials used in this study. Table 1

Group 1: 37% phosphoric acid + Adper Single Bond 2 (ASB2) 

The cavity was etched with 37% phosphoric acid gel (ScotchbondTM Universal Etchant; 3M ESPE, St Paul, MN, USA) for 15 s. After rinsing, the dentin surface was gently dried and left visibly moist. Double adhesive coat was applied, followed by 5 s of gentle air drying. Further, light cure was done for 20 s through a visible light-curing unit (Hilux Expert, Benlioglu, Ankara, Turkey). Prior to this, a curing radiometer was used for testing purpose, which showed an output of 600 mW/cm2 that was considered adequate. A metal matrix system (Tofflemire, Teledyne Water Pik, Ft. Collins, CO, USA) was then fitted onto the prepared tooth before the application of the restorative material. A nanocomposite FiltekTM Ultimate (3M ESPE, St. Paul, MN, USA) was placed in 2 mm increments and polymerized for 20 s per increment.

Group 2: Er:YAG laser conditioning + 37% phosphoric acid + ASB2

Cavity preparation was followed by enamel and dentin surfaces conditioning with Er:YAG laser for 30 s at a wavelength of 2.94 µm, with 250-350 mJ output, 250 to 500 µs pulse duration, 2 Hz frequency and 20 mm distance between the target tissue and lens under constant cooling (5 mL/min) ( Table 2). The laser conditioning was followed by 37% phosphoric acid etching. The adhesive and composite resin applications were performed in a similar manner as in group 1.

**Table 2:**
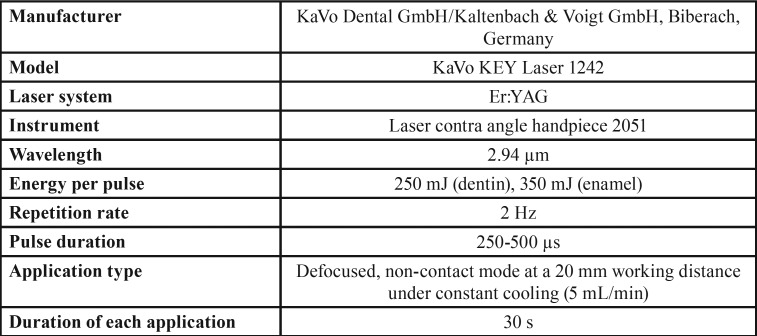
Laser parameters.

Group 3: Clearfil SE Bond (CSE)

The enamel and dentine surfaces were conditioned with primer for 20 s and were gently air dried for 5 s. Then, the adhesive was applied for 15 s using the same applicator, gently air dried for 3 s and light cured for 10 s. The composite resin application was performed in a similar manner as in group 1.

Group 4: Er:YAG laser conditioning + CSE

The laser conditioning was performed similar to that of group 2. The primer, adhesive and composite resin applications were performed in a similar manner as in group 3. 

Group 5: Adper Easy One (AEO)

With 20 s agitation, the application of bonding agent was performed, which was further air dried for 5 s and light cured for 10 s. The composite resin application was performed in a similar manner like that of group 1.

Group 6: Er:YAG laser conditioning + AEO

The laser conditioning was performed similar to that of group 2. The application of adhesive and composite resin was performed in the same manner as in group 5.

Finishing procedures were performed with a fine diamond bur (G & Z Instrumente GmbH, Lustenau, Austria), and then polished with a graded series of Sof-Lex discs (3M ESPE, St. Paul, MN, USA). Cervical margins were preserved against polishing. All groups were assigned to two subgroups according the gingival wall level: 1 mm coronal to the cemento-enamel junction (enamel area; denoted by letter A) or 1 mm apical to the cemento-enamel junction (dentinal area; denoted by letter B)

-Microleakage test

All specimens were stored in distilled water at 37 °C for 24 h, which were thermocycled at 5 °C and 55 °C, 1000 times each with 30 s as the dwell time. At the root apices, composite resin (TPH Spectrum, Dentsply deTrey, Konstanz, Germany) was used to seal the samples. Then nail varnish was applied on the surface of the tooth in two coats below the restoration margins. The specimens were then soaked in 0.5% aqueous basic fuchsin dye for a day. Subsequent rinsing was done with water, and the dye was removed. The specimens were then air dried at room temperature. Longitudinal sections of all samples were obtained through their centers mesial to distal using a water-cooled, slow-speed diamond saw (Isomed, Buehler Ltd, Lake Bluff, IL, USA). The lengths of dye penetration of cervical margins were examined using a stereomicroscope (Leica MS5, Singapore, Singapore) under 40X magnification. It was assessed by two examiners using a four-point scale: grade 0 = no dye penetration; grade 1 = dye penetration up to one-third of the gingival wall; grade 2 = dye penetration more than one third, but less than two-thirds of the gingival wall; and grade 3 = dye penetration more than two-thirds, or the full extent of the gingival wall (8).

-Scanning electron microscope (SEM) evaluation

A sample from each group was considered for adhesive interface analyses by SEM (Zeiss Evo 50; Carl Zeiss, Oberkochen, Germany). These additional samples were not submitted to the microleakage test. The samples were cross-sectioned in half using a diamond disc. The sections were decalcified using 37% phosphoric acid for 15 s and further deproteinized by immersing in 5% NaOCl solution for 2 min. The samples were maintained for 48 h in a desiccator and mounted in stubs. They were then sputter coated with gold and observed under SEM.

-Statistical analysis

Descriptive statistics for ordinal data were expressed as median (interquartile range). The number of cases and percentages were used for categorical data. The differences in leakage scores between groups were compared by Mann Whitney U test. Kruskal-Wallis test was applied for comparing more than two independent groups. Categorical data were analyzed by Likelihood Ratio or Fisher’s exact test, as appropriate. A *p* value < 0.05 was considered as statistically significant. However, for controlling Type I error, the Bonferroni Correction was applied for all possible multiple comparisons.

## Results

**Figure 1:**
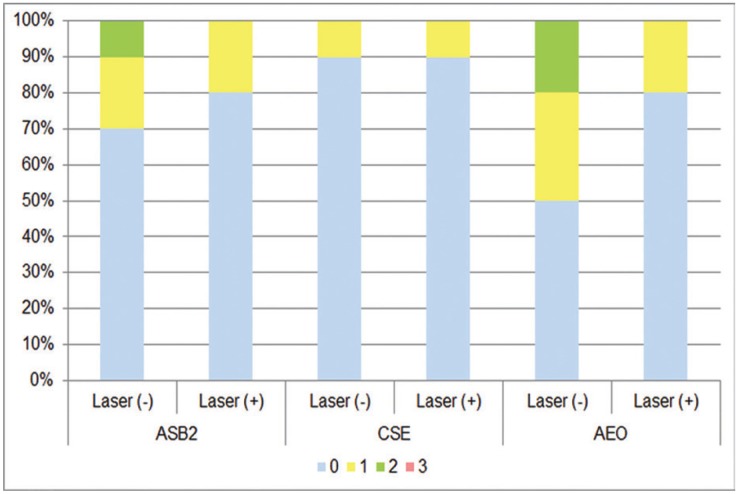
Frequency distributions of dye penetration scores for each group in enamel. ASB2, 37% phosphoric acid + Adper Single Bond 2; CSE, Clearfil SE Bond; AEO, Adper Easy One; Laser (-), conventional conditioning; Laser (+), supplementary laser conditioning.

**Figure 2:**
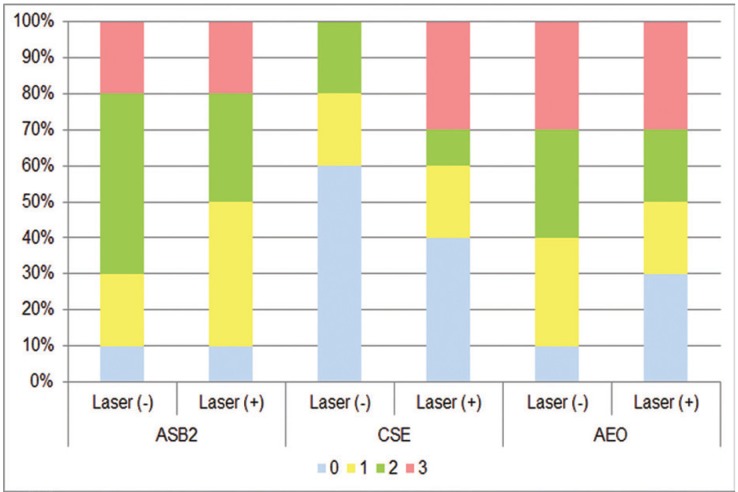
Frequency distributions of dye penetration scores for each group in dentin. ASB2, 37% phosphoric acid + Adper Single Bond 2; CSE, Clearfil SE Bond; AEO, Adper Easy One; Laser (-), conventional conditioning; Laser (+), supplementary laser conditioning.

Figures 1 and 2 show the distribution frequency of dye penetration scores for each group based on the four-point scale in both enamel and dentin. When the dye penetration scores for all groups with the same adhesive system were compared, no significant differences were observed between the conditioning modalities (conventional vs. supplementary laser conditioning) in both enamel and dentin ( Table 3). In line with this, there were also no significant differences between the conditioning modalities when the incidences of microleakage for groups with the same adhesive system were compared in both enamel and dentin ( Table 4). When the dye penetration scores for the groups with the same laser application modality [laser application absent (conventional) and laser application present] were compared, there were no significant differences between the groups in both enamel and dentin ( Table 3). There were also no significant differences between groups when the incidences of microleakage for groups with the same laser application modality were compared in both enamel and dentin ( Table 4). Group 1 (ASB2) (*p* = 0.004) and group 2 (laser + ASB2) (*p* = 0.002) showed significantly reduced dye penetration scores in enamel (denoted as “A”) as compared to the scores obtained from dentin (denoted as “B”). In contrast, no significant difference was observed between the median penetration scores of enamel and dentin in groups 3 (CSE), 4 (laser + CSE), 5 (AEO), and 6 (laser + AEO) ( Table 3). Group 2 showed significantly lower incidence of microleakage in enamel as compared to dentin (*p* = 0.005). No significant differences were observed between the incidence of microleakage detected in enamel and dentin in groups 1, 3, 4, 5, and 6 ( Table 4).

**Table 3:**
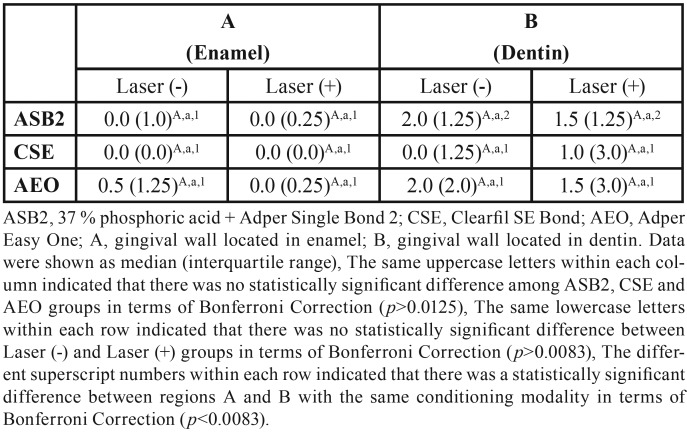
The comparisons of median leakage scores among groups.

**Table 4:**
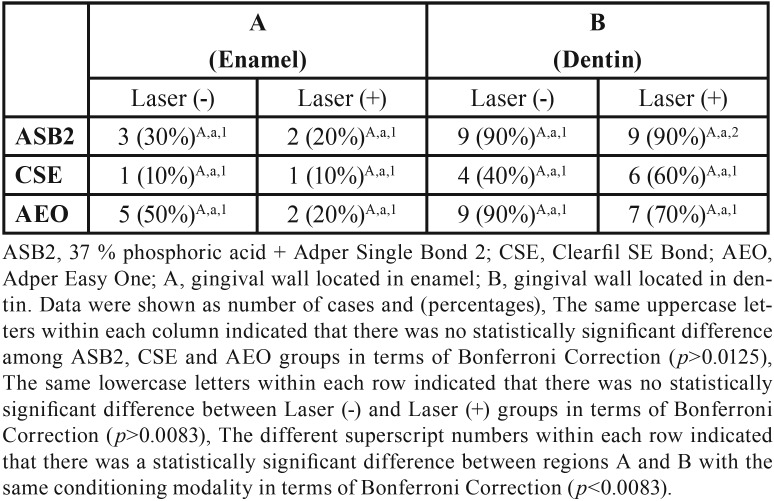
The incidence of microleakage for each group.

**Figure 3:**
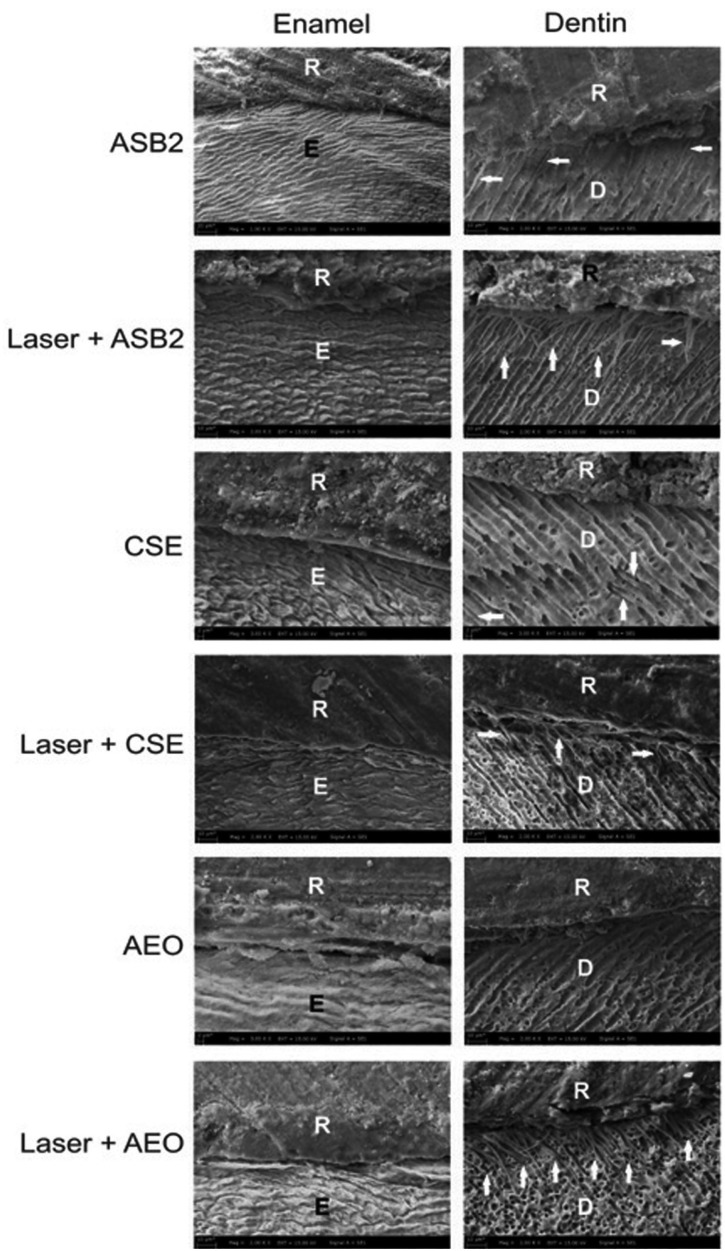
Representative scanning electron microscopic images showing the enamel and dentin interfaces for each group. The arrows indicate resin tags. ASB2, 37% phosphoric acid + Adper Single Bond 2; CSE, Clearfil SE Bond; AEO, Adper Easy One; Laser +, supplementary laser conditioning; D, dentin; E, enamel; R, composite resin.

Figure 3 shows the representative SEM micrographs of the enamel and dentin interfaces with different conditioning modalities and adhesives. Both the specimens from group 1 and group 2 showed a close relationship with the substrate in enamel region. Although resin tags were formed, both specimens could be seen with a noted separation present between the adhesive layer and the dentin surface. For the specimens treated in group 3 and group 4, the dentin showed resin tags. Also, gap-containing areas present between the cement region and dentin were observed in laser + CSE treated specimen. Both specimens from groups 3 and 4 revealed a good adaptation with the enamel. A better interaction with enamel was observed in group 6 compared to group 5. An AEO bonded to dentin micrograph showed a close adaptation with the substrate; however, no resin tags could be detected. Laser + AEO created a uniform resin tag formation in dentin, but the adhesive layer was partly separated from dentin.

## Discussion

Many previous research reports have demonstrated the ability of lasers to modify the enamel and dentin surface showing micro-irregularities, along with absence of smear layer (9-11). The literature presents controversial findings regarding the relationship between the laser applications and sealing ability. Some studies (12-14) have reported that the Er:YAG laser used alone or used in combination with acid-etching technique creates a surface with sealing ability that is similar or better than that produced by non-laser applied counterparts. Other reports (15,16) showed higher dye penetration rates when the margins of the composite restorations were prepared with Er:YAG laser. Arbabzadeh Zavareh *et al.* (17) showed that the use of laser for surface conditioning of enamel margins increased the grade of microleakage in CSE group, whereas it yielded no significant result in terms of CSE’s sealing ability on dentin margins. Our findings are mostly in agreement with the first group of studies and partly consistent with Arbabzadeh Zavareh *et al.*’s study (17) because there seemed to be a comparable sealing ability in the present study when the conventionally-conditioned and laser-conditioned groups with the same adhesive system were compared. Additionally, the present study also indicated that laser conditioning was in favor of ASB2 application in enamel region, which resulted in significantly lower incidence of microleakage as compared to the incidence found in dentin.

In the present investigation, etch-and-rinse adhesive approach showed increased leakage scores in the dentin as compared to the enamel when either of the conditioning methodologies was applied. No statistically significant differences were observed in self-etch adhesive approaches in determining microleakage scores between the enamel and dentin regions. Hence, it can be concluded that dentin bonding is more technique- and substrate-sensitive compared to enamel bonding. In direct adhesive Class II restorations, enamel has proved to be a more reliable substrate compared to dentin because of its homogeneous structure and hydrophobic character (18,19). Therefore, the formation of a consistent bonding with less microleakage can be seen in the case of enamel margins compared to the dentinal margins (20). One of the major reasons behind it can be the heterogeneous nature of the dentin requires the adhesive system to simultaneously accommodate the properties of the smear layer, collagen, hydroxyapatite, fluids, and dentinal tubules (21). Although the present study showed no significant differences between the etch-and-rinse and self-etch approaches in dentinal regions, the self-etch approach has been known to be advantageous on dentinal surfaces (22). This is because the susceptibility of the adhesive to moisture contamination through transudation of dentinal wetness in the case of self-etch adhesives has been reported to be low compared to etch-and-rinse adhesives (23).

Throughout the last decades, self-etch adhesive systems have attained increased popularity. Depending on whether a self-etching primer and bonding steps are supplied separately or are incorporated into a single solution, both the two-step and one-step self-etch adhesives have proved to be advantageous. Both have shown to reduce the application time and technique-related sensitivity (24). On the other hand, there is an ongoing argument with respect to the efficacy of the sealing ability on the enamel surface with self-etch adhesives. Some authors supported the selective enamel etching by means of the supplementary use of phosphoric acid before the application of self-etch adhesive application (25) while certain other researchers claimed that the sealing ability of self-etch adhesives are similar to that of etch-and-rinse adhesives on the enamel margins (26,27). In agreement with the latter studies, the sealing performance of CSE and AEO was comparable with that of the etch-and-rinse adhesive, ASB2, used on enamel margins. These results are in line with a previous study (28) where CSE and an etch-and-rinse adhesive, Single Bond (3M ESPE, Seefeld, Germany), showed similar sealing ability on enamel margins. It can also be noted that there were no significant differences between AEO and laser + AEO in both the regions with respect to dye penetration. SEM observations revealed that supplementary laser conditioning provided a better interaction with both enamel and dentin. In this regard, it would be advisable to perform a laser conditioning before AEO application to ensure the long-term integrity of the bonding process.

## Conclusions

Within the limitation of the present study, comparable sealing performances were found for all adhesive systems in restoring the gingival walls that located in both the enamel and dentin regardless of the previous laser conditioning. ASB2 showed a higher degree of microleakage in dentin versus enamel while both CSE and AEO showed similar sealing abilities between enamel and dentin. The additional Er:YAG laser conditioning may be beneficial before ASB2 application in order to promote the marginal sealing ability in the enamel region. In this regard, the use of CSE and AEO may be considered as an alternative to ABS2 for restoring Class II cavities in endodontically treated teeth due to their simple application procedures.
